# Condition interference in rats performing a choice task with switched variable- and fixed-reward conditions

**DOI:** 10.3389/fnins.2015.00027

**Published:** 2015-02-13

**Authors:** Akihiro Funamizu, Makoto Ito, Kenji Doya, Ryohei Kanzaki, Hirokazu Takahashi

**Affiliations:** ^1^Neural Computation Unit, Okinawa Institute of Science and Technology Graduate UniversityOkinawa, Japan; ^2^Graduate School of Information Science and Technology, The University of TokyoTokyo, Japan; ^3^Research Center for Advanced Science and Technology, The University of TokyoTokyo, Japan

**Keywords:** habit, goal-directed, reinforcement learning, Q-learning, prefrontal cortex, striatum, task switching

## Abstract

Because humans and animals encounter various situations, the ability to adaptively decide upon responses to any situation is essential. To date, however, decision processes and the underlying neural substrates have been investigated under specific conditions; thus, little is known about how various conditions influence one another in these processes. In this study, we designed a binary choice task with variable- and fixed-reward conditions and investigated neural activities of the prelimbic cortex and dorsomedial striatum in rats. Variable- and fixed-reward conditions induced flexible and inflexible behaviors, respectively; one of the two conditions was randomly assigned in each trial for testing the possibility of condition interference. Rats were successfully conditioned such that they could find the better reward holes of variable-reward-condition and fixed-reward-condition trials. A learning interference model, which updated expected rewards (i.e., values) used in variable-reward-condition trials on the basis of combined experiences of both conditions, better fit choice behaviors than conventional models which updated values in each condition independently. Thus, although rats distinguished the trial condition, they updated values in a condition-interference manner. Our electrophysiological study suggests that this interfering value-updating is mediated by the prelimbic cortex and dorsomedial striatum. First, some prelimbic cortical and striatal neurons represented the action-reward associations irrespective of trial conditions. Second, the striatal neurons kept tracking the values of variable-reward condition even in fixed-reward-condition trials, such that values were possibly interferingly updated even in the fixed-reward condition.

## Introduction

The cortico-basal ganglia circuit is involved not only in movement control, but also in inference-, experience- and reward-based decision making (Hikosaka et al., 1999; Daw et al., 2005; Cohen et al., 2007; Doya, 2008; Ito and Doya, 2011). Many anatomical and functional studies suggest that this diverse set of functions is simultaneously implemented in parallel in the circuit [anatomy: (Haber, 2003; Voorn et al., 2004; Gruber and McDonald, 2012); function: (Tanaka et al., 2004; Balleine et al., 2007; Yamin et al., 2013)]. A typical example of this parallel circuit is the neural implementation of response-outcome (R-O) and stimulus-response (S-R) associations: the former association is driven by the medial part of the circuit, including the prelimbic cortex and the dorsomedial striatum, for producing flexible learning behaviors (Corbit and Balleine, 2003; Yin et al., 2005a,b), while the latter association is implemented in the infralimbic cortex and the dorsolateral striatum to execute inflexible behaviors (Yin et al., 2004; Balleine and Killcross, 2006).

In the parallel decision-making circuits, humans and animals select actions in various situations. The abilities to anticipate and store outcomes of options in any situation are crucial. Despites its importance in action learning, decision processes and neural substrates involved in various situations are still unclear, partly because behavioral experiments have usually been designed to eliminate situational effects as far as possible, for the sake of simplicity. These past studies may hypothesize that outcome estimation in each condition is independently processed; however, humans often cannot perform two tasks at once without interference (Monsell, 2003). This task-switching cost predicts that the cortico-basal ganglia circuit contains some conditional interferences.

Decision processes in the cortico-basal ganglia circuit are theoretically explained by the reinforcement learning framework (Corrado and Doya, 2007; O'Doherty et al., 2007; Doya, 2008). The framework has two steps for decisions: value updating, in which agents update the expected rewards (i.e., values) with past actions and rewards, and action selection, in which agents select actions based on the values (Sutton and Barto, 1998). Although task conditions are considered independently in classical reinforcement learning theories, we hypothesize that decision making under various conditions leads to some interference among conditions in value updating and/or action selection. Especially when interference occurs in value updating, its neural correlates may be observed in the striatum, because the striatum is known to represent and store action values (Samejima et al., 2005; Lau and Glimcher, 2008).

Using rats, we conducted a choice task with a random trial sequence of variable- and fixed-reward conditions to test whether rats had condition interference. Variable- and fixed-reward conditions were designed to investigate flexible and inflexible behaviors, respectively; reward probabilities in the variable-reward condition varied between blocks of trials, while they were fixed in the fixed-reward condition. Neural activities of the prelimbic cortex and dorsomedial striatum (i.e., a candidate flexible-behavior network) were electrophysiologically recorded to investigate neural substrates of condition interference. We used rats because their parallel cortico-basal ganglia circuits for decision making are well examined and established (Voorn et al., 2004; Balleine, 2005). Results of this study from reinforcement learning models suggested that, although rats distinguish the trial conditions, they update values in a condition-interference manner. Some striatal neurons represented values required for the variable-reward condition even during fixed-reward-condition trials, suggesting that these representations caused the condition interference between flexible and inflexible behaviors.

## Materials and methods

All procedures were approved by the institutional committee at the University of Tokyo and performed in accordance with the “Guiding Principles for the Care and Use of Animals in the Field of Physiological Science” of the Japanese Physiological Society. We used five male Long-Evans rats (240–380 g); two rats performed both the behavioral and electrophysiological experiments, and the remaining three rats performed only the behavioral experiments. Food was provided after the task to maintain animal body weight at no less than 85% of the initial level. Water was supplied freely.

### Behavioral task

**Figure 1:**
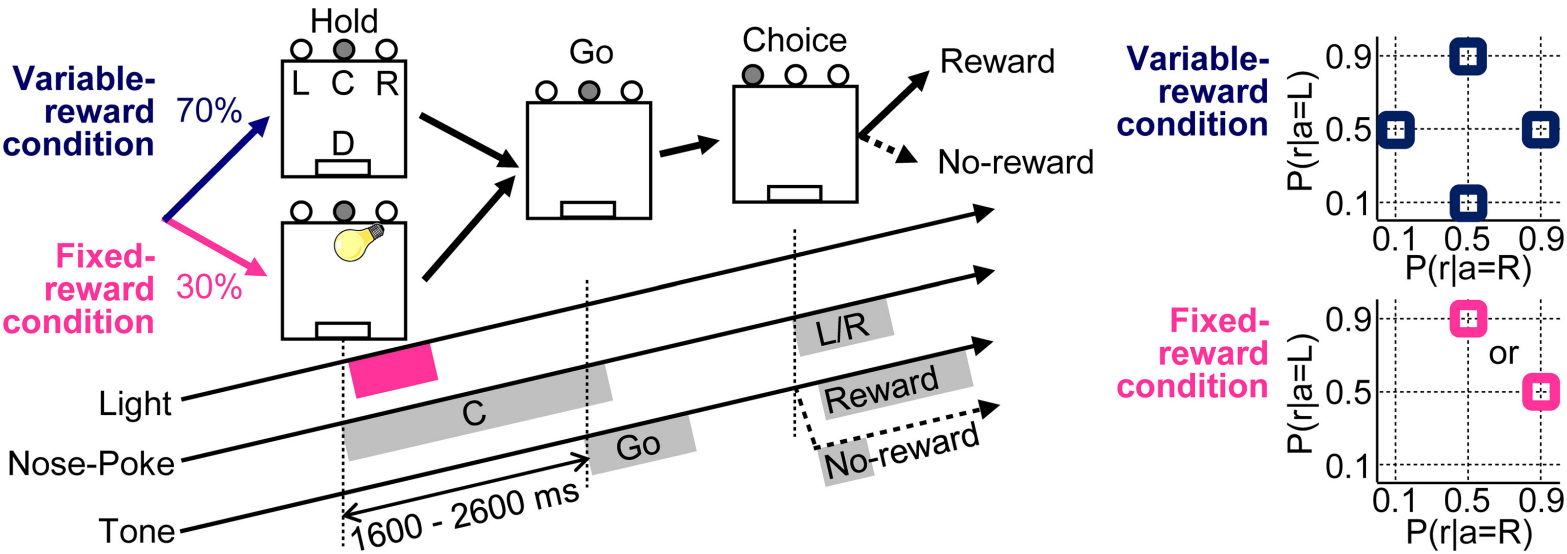
**Free choice task**. Variable- and fixed-reward conditions were randomly assigned for each trial in a 70% / 30% ratio, respectively. In both conditions, each trial was initiated when a rat poked its nose into the center hole (C), after it had to keep poking for 1600–2600 ms until a “go” tone sounded (Hold). During the fixed-reward condition only, a short light stimulus was presented during the center-hole poking to inform rats that the trial was a fixed-reward-condition trial. After the presentation of “go” tone (Go), rats had to choose either a left (L) or a right (R) hole, and a reward of a food pellet was dispensed stochastically (D) (Choice). In the variable-reward condition, reward probabilities were selected randomly (90–50%, 50–10%, 10–50%, and 50–90% for left-right) and the reward setting changed based on the choice performance of rat. In the fixed-reward condition, the reward probability was constant with either 90–50% or 50–90% for all the sessions, and the reward setting was pre-determined for each rat.

All experiments were conducted in a 36 × 36 × 37 cm experimental chamber (O'Hara & Co. Ltd.) placed in a sound-attenuating box (Funamizu et al., 2012). The experimental chamber had three nose-poke holes on one wall and a pellet dish on the opposite side of the chamber (Figure 1). Four light emitting diode (LED) high-intensity lamps (white) were placed above the center hole for light stimuli. A speaker was placed above the chamber for sound stimuli. All durations of poking, presence, and consuming of pellets were captured with infrared sensors and were recorded with a sampling rate of 1 kHz (Cyberkinetics Inc.; Cerebus Data Acquisition System).

Our task had variable- and fixed-reward conditions; one of the conditions was randomly assigned for each trial with proportion of 70% and 30%, respectively (Figure 1). Only in fixed-reward-condition trials, a light stimulus was presented to inform rats of the trial condition. In each trial, rats first performed a nose-poke in the center hole, and they continued poking until a “go” tone with a frequency of 5 kHz, an intensity of 50 dB SPL (sound pressure level in decibels with respect to 20 μ Pa) and a duration of 500 ms was presented. In the fixed-reward condition, a light stimulus was presented for about 600 ms immediately after the initiation of center-hole poking. If rats failed to continue poking until the presentation of the “go” tone, an error tone was presented (1 kHz, 70 dB SPL, 50 ms), and the trial was scored as an error. After the presentation of “go” tone, rats selected either the left or right hole within 15 s and received a reward of a food pellet (25 mg), presented stochastically. A reward tone (20 kHz, 70 dB SPL, 2000 ms) was presented immediately after the choice in a rewarded trial. In contrast, a no-reward tone (1 kHz, 70 dB SPL, 50 ms) was presented in a non-rewarded trial. If rats did not select choices within 15 s from the presentation of the “go” tone, the error tone was also presented, as in the error trial.

In the variable-reward condition, the reward probability of each choice changed among four settings: 90–50%, 50–90%, 50–10%, and 10–50% in regard to left-right choices. Variable-reward-condition trials with the same reward-probability setting were referred to as a block; a block consisted of at least 20 trials. Subsequently, the block was changed when the rat selected the more rewarding hole in ≥80% of the last 20 variable-reward-condition trials (Ito and Doya, 2009; Funamizu et al., 2012). The block change was conducted so as to (i) include all four reward-probability settings in each of the four blocks and (ii) not to repeat any of the settings. Each rat performed at least four blocks per day (i.e., per session) and any sessions consisting of fewer than five blocks were excluded from the analysis.

In the fixed-reward condition, the reward probability was constant in all sessions, and was set to either 90–50% or 50–90% in the left-right choices for each rat. Each rat selected the more-rewarding choice more than 80% through a session in fixed-reward condition, and any sessions in which rats failed to select the optimal choice were not used in the analysis.

Thus, our task required the rats to select the more-rewarding hole ≥80% of the time in both variable- and fixed-reward conditions. Therefore, the rats needed to distinguish the trial type in order to achieve the 80% correct-choice criterion, when the more-rewarding holes of variable- and fixed-reward conditions were different.

In both the variable- and fixed-reward conditions, we provided an extinction phase which never presented a reward for choices in a random sequence of five variable-reward-condition trials and five fixed-reward-condition trials (i.e., successive 10 trials in total) to characterize the behaviors in variable- and fixed-reward conditions. The extinction phase was conducted after the reward probability of variable-reward-condition block was identical to that of fixed-reward condition. In the extinction phase, we investigated the sensitivity to this treatment from the choice preferences of rats. Flexible or inflexible behaviors should change or retain choices with the outcome extinction, respectively.

### Surgery

After rats practiced the free choice task, they were anesthetized with sodium pentobarbital (50 mg/kg, i.p.) and placed in a stereotaxic frame (Narishige). Atropine sulfate (0.1 mg/kg) was also administered at the beginning of the surgery to reduce the viscosity of bronchial secretions (Takahashi et al., 2011; Funamizu et al., 2013). The cranium and dura over recording sites were removed and four small craniotomies were conducted for anchoring screws. The screws were used for the ground electrode in electrophysiology. Two drivable parallel electrode bundles were inserted into the prelimbic cortical site in the right hemisphere (2.5 mm in anterior-posterior (AP) and 0.55 mm in medio-lateral (ML) from the bregma with a depth of 2.5 mm from the surface of brain). The three electrode bundles were inserted into the dorsomedial striatum site in the right hemisphere (0.2 mm in AP, 2.0–3.0 mm in ML with a depth of 3.4 mm) (Stalnaker et al., 2010; Wang et al., 2013). Each electrode bundle was lowered 125 μm after each session such that we could get new neurons in every session (Ito and Doya, 2009). The bundle was composed of seven or eight Formvar-insulated nichrome wires with the bare diameter of 25 μm (A-M Systems). The wires were inserted into a stainless-steel guide cannula with an outer diameter of 0.3 mm. The tip of each wire was electroplated with gold to obtain an impedance of 100–200 kΩ at 1 kHz. In total, five electrode bundles were inserted in the brain, and 14 and 24 wires were inserted in the prelimbic cortex and dorsomedial striatum, respectively.

### Electrophysiological recording

During the choice task, recorded neural signals were amplified and stored with a 62-ch multiplexer neural-recording system (Triangle biosystems international; TBSI) and a Cerebus data acquisition system (Cyberkinetics Inc.) with an amplified gain of 1000, a band-pass filter of 0.3–7500 Hz, and a sampling frequency of 30 kHz. We then applied an offline digital high-pass filter of 200 Hz (Matlab; The Mathworks). When the signal became below or above its root mean square (RMS) times 5.5, the signal was defined as spike activity (Torab et al., 2011). Offline spike sorting was conducted using Spike 2 (CED), with which spike waveforms were classified into several groups based on template matching. Groups of waveforms that appeared to be action potentials were accepted, while all others were discarded.

### Histology

After electrophysiological recording, rats were anesthetized with sodium pentobarbital (50 mg/kg, i.p.), and a positive current of 10 μ A was passed for 10–20 s through one or two electrodes of each bundle to mark the final recording positions (Ito and Doya, 2009). Rats were perfused with 10% formalin containing 3% potassium hexacyanoferrate (II), and the brain was carefully removed from the cranial bone. Sections were cut at 90 μm with a vibratome (DTK-2000, D.S.K.) and stained with cresyl violet. The position of each recorded neuron was estimated from the final position and the distance that the bundle was moved. If the position was outside the prelimbic cortex or dorsomedial striatum, the data were discarded.

### Behavioral analysis

In the analyses of behaviors during the choice task, error trials (in which rats failed to keep poking in the center hole, or took more than 15 s to select the left or right hole) were removed, and the remaining sequences of successful trials (in which rats successfully made a left or right choice) were used.

#### Model-free analysis

We first analyzed choice preferences during the extinction phase to identify whether rats had flexible or inflexible behaviors in the variable- and fixed-reward conditions. We then assessed the interference of variable- and fixed-reward conditions in the choice behaviors. We compared conditional choice probabilities between two trial sequences: repeated sequences [e.g., variable-reward-condition trial to variable-reward-condition trial (Var. – Var.)], in which probabilities were calculated based on the action-outcome experience in the last trial with a same condition; and interleaved sequences (e.g., Var. – Fix. – Var.), in which probabilities were calculated based on the experience in the next-to-last trial with the same condition, so that the last different-condition trial was ignored (**Figure 4Bi**). If the choice of each condition was independently learned and the interleaved trial caused no interference, conditional probabilities in the two trial sequences became the same.

#### Model-based analysis

We analyzed choice behaviors of rats with reinforcement learning models and a fixed-choice model to test (i) whether interference occurred in choice learning, and (ii) whether it occurred in the value updating or action selection phase. We denoted the action as *a* ∈ [*L* (left), *R* (right)], the reward as *r* ∈ [1, 0] and the condition as *C* ∈ [*V* (variable), *F* (fixed)]. We assumed that rats predicted the expected reward of each choice (i.e., action value) in each condition, *Q*_*a*, *C*_: rats had four action values in total. A choice probability was predicted with the following soft-max equation based on the action values:

(1) $$\begin{array}{l} P\left(a\left(t\right)=L\right)=\\ \frac{1}{\begin{array}{l} 1+exp\left[Q_{R,C(t)}(t)-Q_{L,C(t)}(t)+\;G_{C(t)}\text{​}\left\{Q_{R,\bar{C}}\left(t\right)-Q_{L,\bar{C}}\left(t\right)\right\}\text{​}\right]\text{​} \end{array}}, \end{array}$$

where *C*(*t*) and *C* were trial and non-trial conditions, i.e., *C* ≠ *C*(*t*); for example, when the presented trial was a variable-reward condition, *C* was a fixed-reward condition. *G*_*C*(*t*)_ was a free parameter depending on the trial condition. This parameter adjusted the contribution of action values of a non-trial condition in the choice prediction.

A fixed-choice model had the action value as a free parameter, assuming a constant value in all trials:

(2) $$\{\begin{array}{l} \begin{array}{l} Q_{R,C}=q_{C}\\ Q_{L,C}=1-q_{C} \end{array} \end{array},$$

where *q_C_* was a free parameter depending on the value condition. If the fixed-choice model fit a choice behavior, the behavior had no-learning and no condition-interference in value updating.

**Figure 2:**
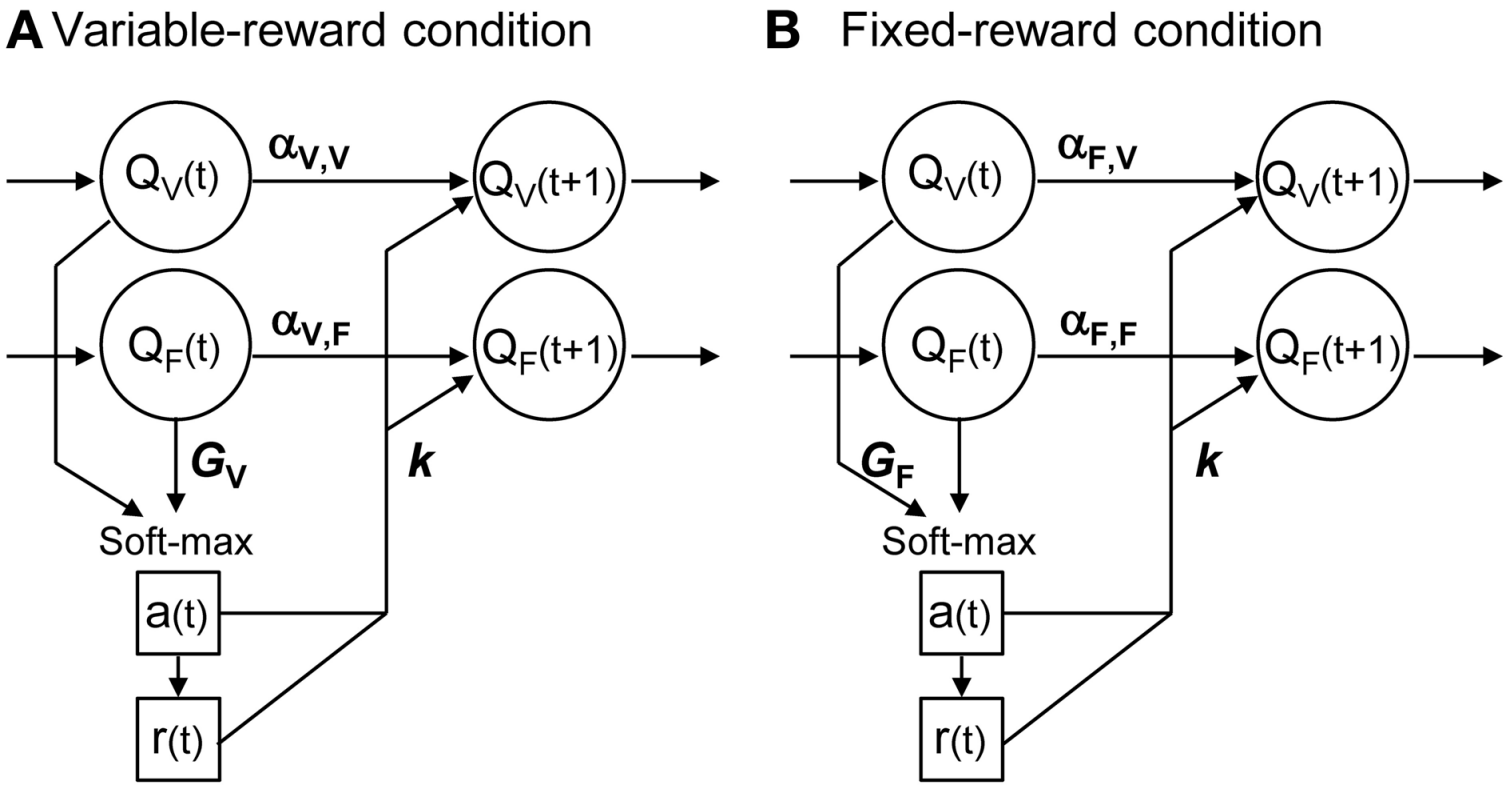
**Interference reinforcement learning model**. Our reinforcement learning models assumed that rats estimated expected rewards (values) of left and right choices in both variable- and fixed-reward conditions, i.e., *Q_V_* and *Q_F_*; models had four action values in total. All action values were updated both in variable-reward-condition **(A)** and fixed-reward-condition **(B)** trials. α was the learning rate or forgetting rate in the selected or unselected option, respectively; α depended on the trial condition and value condition. *k* was the reinforcer strength of the outcome. A choice probability was predicted with a soft-max equation based on all values. The soft-max equation had a free parameter, *G*, which adjusted the contribution of action values from the non-trial condition in the choice prediction.

Figure 2 shows the scheme of proposed reinforcement learning models. We updated the action value in each condition, *Q*_*a*, *C*_, in accordance with Ito and Doya (2009):

(3) $$\begin{array}{l} Q_{a,V}\left(t+1\right)=\\ \left\{ \begin{array}{l} \left(1-\alpha_{1,C\left(t\right),V}\right)Q_{a,V}\left(t\right)+\alpha_{1,C\left(t\right),V}k_{1}\;if\;a=a\left(t\right),\;r\left(t\right)=1\\ \left(1-\alpha_{1,C\left(t\right),V}\right)Q_{a,V}\left(t\right)-\alpha_{1,C\left(t\right),V}k_{2}\;if\;a=a\left(t\right),\;r\left(t\right)=0\\ \left(1-\alpha_{2,C\left(t\right),V}\right)Q_{a,V}\left(t\right)\;if\;a\neq a\left(t\right) \end{array}\right.\\ Q_{a,F}\left(t+1\right)=\\ \left\{ \begin{array}{l} \left(1-\alpha_{1,C\left(t\right),F}\right)Q_{a,F}\left(t\right)+\alpha_{1,C\left(t\right),F}k_{1}\;if\;a=a\left(t\right),\;r\left(t\right)=1\\ \left(1-\alpha_{1,C\left(t\right),F}\right)Q_{a,F}\left(t\right)-\alpha_{1,C\left(t\right),F}k_{2}\;if\;a=a\left(t\right),\;r\left(t\right)=0\\ \left(1-\alpha_{2,C\left(t\right),F}\right)Q_{a,F}\left(t\right)\;if\;a\neq a\left(t\right), \end{array}\right. \end{array}$$

where *a*(*t*), *r*(*t*) and *C*(*t*) were the action, reward, and condition at trial *t*, respectively. Action values of both variable- and fixed-reward conditions were updated every trial, irrespective of the trial condition. α_1_, α_2_, *k*_1_, and *k*_2_ were free parameters. α_1_ showed the learning rate in the chosen option, and α_2_ showed the forgetting rate in the un-chosen option. *k*_1_ and *k*_2_ indicated the strengths of reinforcers in reward and non-reward outcomes, respectively. α_1_ and α_2_ depended on the trial condition, *C*(*t*), and the action-value condition, *C*, to capture differences in (i) learning of variable- and fixed-reward conditions, and (ii) learning by its own condition and by the other condition. Equation (3) had 10 parameters in total.

Equation (3) could take a variety of updating rules by selecting utilized parameters, so that updating rules for the values of variable- and fixed-reward conditions (the upper and lower part of Equation 3, respectively) could be different. When we set α_2_ = *k*_2_ = 0, the equation became a standard Q-learning (Q-learning) (Watkins and Dayan, 1992; Sutton and Barto, 1998). We referred to the equation with α_1_ = α_2_ as a forgetting Q-learning (FQ-learning), and we referred to the full-parameter equation as a differential forgetting Q-learning (DFQ-learning) (Ito and Doya, 2009).

When we set α_1, *C*, *C*_ = α_2, *C*, *C*_ = 0 where *C* ≠ *C* in value updating (Equation 3) and *G_C_* = 0 in action selection (Equation 1), the equations deal with variable- and fixed-reward conditions independently; we referred to the model as an independent model. When we set α_1, *C*, *C*_ = α_2, *C*, *C*_ = 0 in Equation (3), the model independently updated action values of each condition, but interferingly predicted the choices; we referred to it as an action interference model. Also, when we set *G_C_* = 0 in Equation (1), the model interferingly updated action values of the variable- and fixed-reward conditions; we referred to it as a learning interference model.

Initial action values for reinforcement learning models were 0.5 in the left and right choices of the variable-reward condition (i.e., the average reward probability of the four reward-probability settings), and were 0.9 and 0.5 in the optimal and non-optimal choices of the fixed-reward condition.

#### Model comparison

We employed the normalized likelihood to test how well the models fit the choice behaviors of rats (Ito and Doya, 2009; Funamizu et al., 2012). The normalized likelihood, *Z*, was defined as follows:

(4) $$Z={\left[\prod_{t\;=\;1}^{N}z(t)\right]}^{\frac{1}{N}},$$

where *N* and *z*(*t*) were the number of trials and the likelihood at trial *t*, respectively. The likelihood, *z*(*t*), was defined as follows, with the predicted left choice probability *P*(*a*(*t*) = *L*):

(5) $$z\left(t\right)=\left\{ \begin{array}{l} P\left(a\left(t\right)=L\right)\;if\;a\left(t\right)=L\\ 1-P\left(a\left(t\right)=L\right)\;if\;a\left(t\right)=R \end{array}\right..$$

We conducted a 2-fold cross validation for model comparison. In the cross validation, all sessions analyzed were divided into two equal groups. One group provided the training data, and the other group provided the validation data. The free parameters of each model were determined such that the normalized likelihood of the training data was maximized. With the determined parameters, the normalized likelihood of each session in the validation data was analyzed. Then, we switched the roles of the two datasets and repeated the same procedure to obtain normalized likelihoods in all sessions. Cross-validation analysis implicitly took into account the penalty of the number of free parameters (Bishop, 2006).

### Neural analysis

Striatal neurons have often been classified into phasically and tonically active neurons (Lau and Glimcher, 2008; Kim et al., 2009); however, our recording could not find clear criteria to support the classification, partly because the number of neurons recorded was too small. The following analyses were performed without the classification.

To test how neural activities in the prelimbic cortex and dorsomedial striatum were modulated during the task, we employed a stepwise multiple regression analysis (Matlab; Mathworks). Regression analysis was used to investigate neural correlates with actions, rewards, conditions, and associations. The analysis also detected neural correlates with the variables in a reinforcement learning model (Samejima et al., 2005; Ito and Doya, 2009). When the analysis was applied sequentially with a time window of 600 ms, advanced with a time step of 300 ms, we could capture the temporal dynamics of neural coding (Kim et al., 2009; Sul et al., 2011). The regression analysis was defined as follows:

(6) $$\begin{array}{l} y\left(t\right)=\beta_{0}+\beta_{1}C\left(t\right)+\beta_{2}a\left(t\right)+\beta_{3}r\left(t\right)+\beta_{4-23}X\left(t\right)\\ \;+\;\beta_{24-28}M_{C\;=\;C\left(t\right)}\left(t\right)+\beta_{29-33}M_{C\;=\;V}\left(t\right)\\ \;+\;\beta_{34}C\left(t-1\right)+\beta_{35}a\left(t-1\right)+\beta_{36}r\left(t-1\right)\\ \;+\;\beta_{37}T\left(t\right), \end{array}$$

where β_0–37_ were regression coefficients. *y*(*t*) was a spike count with a time window of 600 ms at trial *t*. *C*(*t*), *a*(*t*), and *r*(*t*) were the trial condition (a dummy variable of 1 or −1 for the variable- or fixed-reward condition, respectively), action (1 or −1 for the right or left choice), and reward (1 or −1 for the reward or non-reward outcome) at trial *t*, respectively. These variables at trial *t* − 1 were also included in the regression analysis as *C*(*t* − 1), *a*(*t* − 1), and *r*(*t* − 1). *X*(*t*) showed their interactions [i.e., *C*(*t*) × *a*(*t*), *C*(*t*) × *r*(*t*), *a*(*t*) × *r*(*t*), *C*(*t*) × *a*(*t*) × *r*(*t*)] with a dummy variable of 1 or −1; each interaction had 4, 4, 4, and 8 combinations, and the total was 20 combinations. When a neuron represented at least one combination of each interaction, we defined the neuron as interaction- or association-coding neuron. For example, when a neuron represented a combination of action and reward, i.e., *a*(*t*) × *r*(*t*), we defined the neuron as action-reward association coding. *M*_*C* = *C*(*t*)_ were the five model variables for the presented-trial condition, consisting of the action values (*Q*_*L*, *C*(*t*)_, *Q*_*R*, *C*(*t*)_), state value [*P*(*a*(*t*) = *L*) × *Q*_*L*, *C*(*t*)_ + (1 − *P*(*a*(*t*) = *L*)) × *Q*_*R*, *C*(*t*)_], chosen value (*Q*_*a*(*t*), *C*(*t*)_) and policy (*Q*_*L*, *C*(*t*)_ − *Q*_*R*, *C*(*t*)_) (Lau and Glimcher, 2008; Ito and Doya, 2009; Sul et al., 2011). *M*_*C* = *V*_ were also model variables, but for the variable-reward condition. *M*_*C* = *V*_ were assumed to be tracked both in the variable-reward-condition and fixed-reward-condition trials in our reinforcement learning models (Equation 3). In contrast, values for the fixed-reward condition did not appear in the regression analysis, because the values were turned out to be constant and were difficult to capture with the analysis (see Results). *T*(*t*) was the trial number for detecting a slow drift of firing rate. When Equation (6) had significant regression coefficients (two-sided Student's *t*-test, *p* < 0.01), the neuron was defined as encoding the corresponding variables. In the model variables (i.e., *M*_*C* = *C*(*t*)_ and *M*_*C* = *V*_), we could not get enough neurons encoding each individual variable, because of our sparse recording. Thus, we defined neurons as value coding when they encoded at least one of the five model variables. Model variables were derived from the proposed reinforcement-learning model in which free parameters were set to achieve the maximum likelihood in each session.

First, to investigate neural correlates of actions (i.e., responses: R), rewards (i.e., outcomes: O) and R-O associations, regression analysis was conducted only with neural activities during variable-reward-condition trials. By reducing the condition terms at trial *t*, Equation (6) became as follows:

(7) $$\begin{array}{l} y\left(t\right)=\beta_{0}+\beta_{1}a\left(t\right)+\beta_{2}r\left(t\right)+\beta_{3\text{−}6}X\left(t\right)+\beta_{7\text{−}11}M_{C\;=\;V}\left(t\right)\\ \;+\;\beta_{12}C\left(t-1\right)+\beta_{13}a\left(t-1\right)+\beta_{14}r\left(t-1\right)+\beta_{15}T\left(t\right). \end{array}$$

Second, to investigate neural correlates of conditions (i.e., stimuli: S) and S-O associations, we extracted trials in which rats selected the optimal side of fixed-reward condition. By focusing on the optimal side, we excluded a potential bias caused by the choice asymmetry in the fixed-reward condition in which rats mainly selected the optimal side. By reducing the action terms at trial *t*, Equation (6) became as follows:

(8) $$\begin{array}{l} y\left(t\right)=\beta_{0}+\beta_{1}C\left(t\right)+\beta_{2}r\left(t\right)+\beta_{3\text{−}6}X\left(t\right)+\beta_{7\text{−}10}M_{C\;=\;C\left(t\right)}\left(t\right)\\ \;+\;\beta_{11\text{−}14}M_{C\;=\;V}\left(t\right)+\beta_{15}C\left(t-1\right)+\beta_{16}a\left(t-1\right)\\ \;+\;\beta_{17}r\left(t-1\right)+\beta_{18}T\left(t\right). \end{array}$$

In Equation (8), model variables had 4 terms because the chosen value became identical to the action value in either a left or right choice. Third, to investigate value-coding neurons, the regression analysis of Equation (6) was applied to neural activities in all trials. Value-coding neurons were also investigated in fixed-reward-condition trials; in this case, Equation (7) was applied for fixed-reward-condition trials.

## Results

### Behavioral analysis

#### Model-free analysis

**Figure 3:**
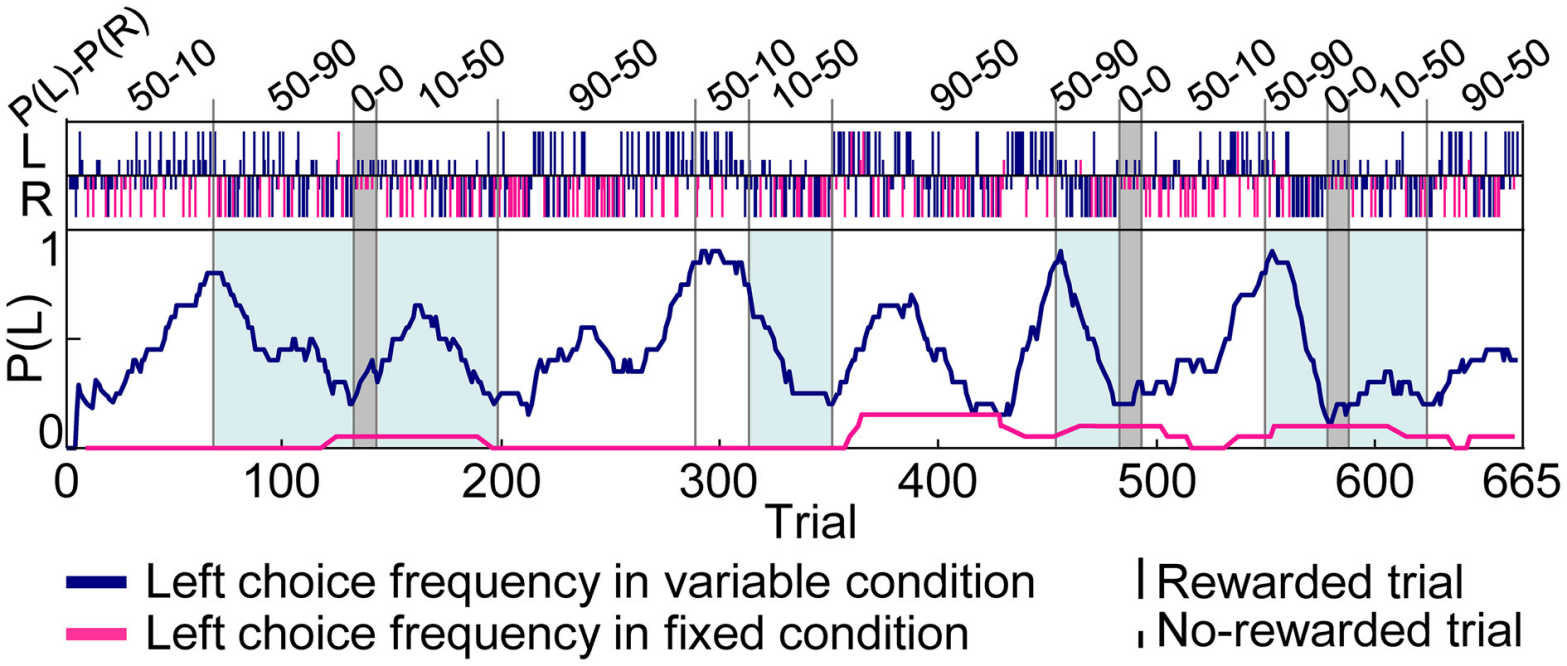
**Example of choice behaviors**. Vertical bars in the upper portions of the inset indicate the left (L) and right (R) choice in each trial. Tall and short bars show rewarded and non-rewarded trials, respectively. Dark blue and pink bars indicate trials with variable- and fixed-reward conditions and lines in the center indicate the left-choice frequency of a given rat in the last 20 trials. The reward probability of the fixed-reward condition was 50–90% for the left-right choice. The reward-probability setting of variable-reward-condition block is shown at the top. In blocks with a light-blue color, more-rewarding choices of variable- and fixed-reward conditions were identical. Rats succeeded in distinguishing the variable- and fixed-reward conditions for action learning.

Figure 3 shows an example of choice behaviors of a rat. In variable-reward-condition trials, the rat changed choices depending on the current setting of reward probabilities to successfully select the better rewarding option. In fixed-reward-condition trials, on the other hand, the rat exhibited fixed behaviors and made the optimal choice more than 80% of the trials. In total, this study analyzed 111 sessions of behavioral data (rat1, 9 sessions; rat2, 21 sessions; rat3, 39 sessions; rat4, 2 sessions; rat5, 40 sessions). Rats underwent an average of 5.57 ± 0.223 blocks (mean ± standard error, here and hereafter) for each session, and required 53.1 ± 1.48 trials in each block to select a more-rewarding choice ≥80% of variable-reward-condition trials. In detail, when the more-rewarding choices of variable- and fixed-reward conditions were identical (e.g., the blocks with a light-blue color in Figure 3), the number of trials per block was 33.5 ± 1.65 and 50.9 ± 2.50 in the high (50–90%) and low (10–50%) reward probabilities, respectively; on the other hand, when the more rewarding choices of variable- and fixed-reward conditions were different, the number of trials was 54.5 ± 3.27 and 75.9 ± 3.31 trials, which were significantly larger than those when the more-rewarding choices of two conditions were identical (Mann–Whitney *U*-test, *p* = 1.12E-8 and 1.06E-8 in the high and low reward probabilities). Thus, the speed to find the better rewarding choice in variable-reward condition depended on the optimal side of fixed-reward condition, suggesting that there was some behavioral interference between the two conditions.

**Figure 4:**
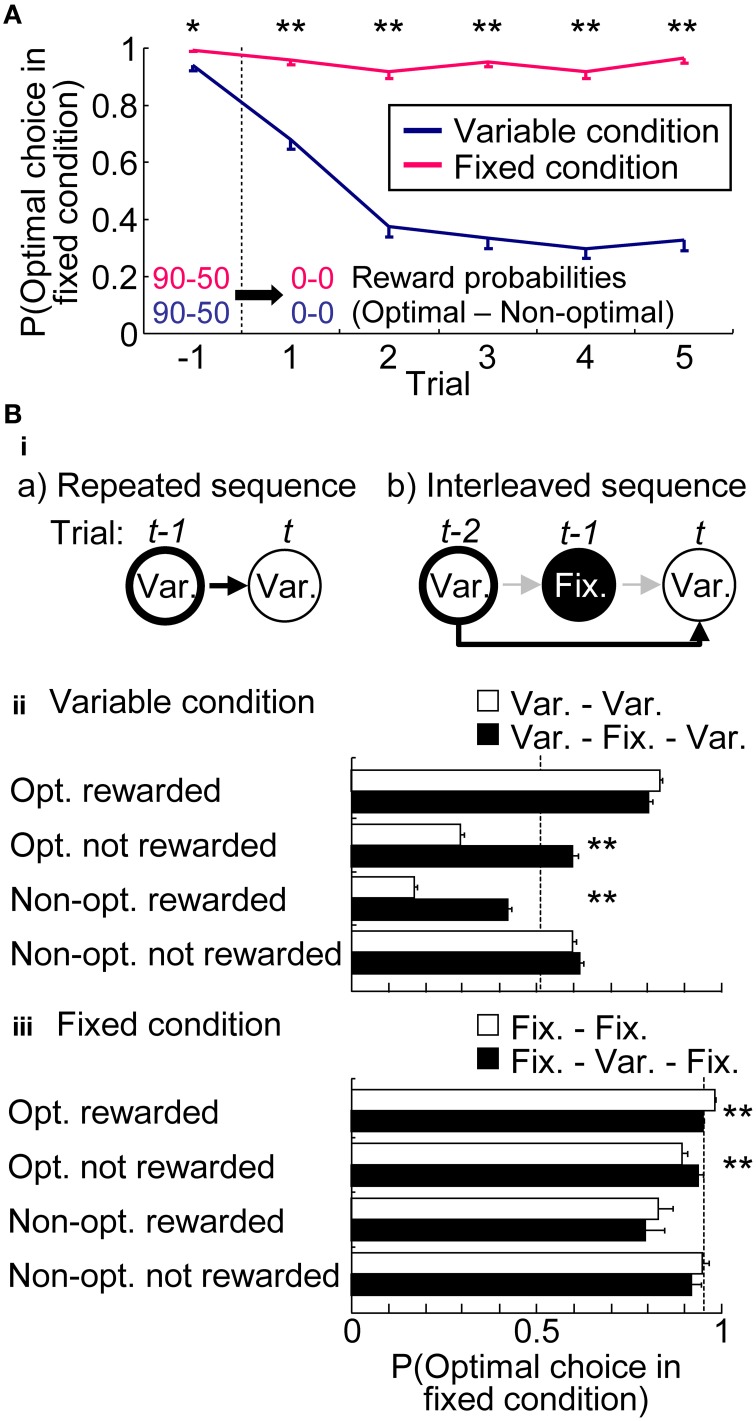
**Choices in variable- and fixed-reward conditions. (A)** Extinction phase. Probabilities of the optimal choice were quantified before and during extinction-phase trials, which were introduced in the variable- and fixed-reward conditions. Means and standard errors are shown. Before the extinction phase, reward probabilities of variable- and fixed-reward conditions were identical: ^*^p < 0.05; ^**^p < 0.01 in a Mann–Whitney *U*-test. **(Bi)** Example of a conditional-probability calculation in repeated (a) and interleaved sequences (b). Depending on the action-outcome experience at trial *t*-1 for (a) and trial *t*-2 for (b), the conditional probability at trial *t* was analyzed. In these examples, the conditional probability of variable-reward-condition trial (Var.) was analyzed, based on the action-outcome experience in the last and the next-to-last trial with a variable-reward condition in (a) and (b), respectively. In (b), the experience in the interleaved trial *t*-1 with the fixed-reward condition was ignored. Action-outcome experiences had 4 types: optimal choice rewarded (Opt. rewarded); optimal choice not rewarded (Opt. not rewarded); non-optimal choice rewarded (Non-Opt. rewarded); non-optimal choice not rewarded (Non-opt. not rewarded). If the choices of variable- and fixed-reward conditions were independently learned, the conditional probabilities of repeated and interleaved sequences became the same. **(ii,iii)** Comparison of conditional probabilities in variable- **(ii)** and fixed-reward condition **(iii)**. Conditional probabilities of making a choice to the optimal side of fixed-reward condition were compared between repeated (white bars) and interleaved sequences (black bars). Means and standard errors of probabilities are shown. Dotted line shows the average choice probability. White and black bars indicate significant differences under some action-outcome experiences, meaning that the interleaved trial interferingly affected the choices: ^**^p < 0.01 in a Mann–Whitney *U*-test.

Figure 4A characterized the choice preferences in the extinction phase. Extinction phase consisted of a random trial sequence of five variable-reward-condition trials and five fixed-reward-condition trials; Figure 4A showed the choices in each condition separately. In the fixed-reward condition, rats continued to select the optimal choice, even after reward omission. In sharp contrast, in the variable-reward condition, rats quickly changed choices (Mann–Whitney *U*-test, *p* = 3.30E-11 − 1.96E-34). This result indicated that rats had flexible and inflexible behaviors in variable- and fixed-reward conditions, respectively.

If rats had condition interference, choices in one condition were affected with action-outcome experiences in the other condition. Figure 4B compared conditional choice probabilities between the repeated sequences and interleaved sequences to test whether the last different-condition trial in interleaved sequences interferingly affected the choices. In variable-reward condition (Figure 4Bii), we found that the interleaved fixed-reward-condition trial significantly shifted the rats' choices to the optimal side of fixed-reward condition in 2 out of 4 action-outcome experiences (Mann–Whitney *U*-test, *p* = 1.58E-31 and 9.47E-30). Although choices in the fixed-reward condition were also significantly affected by the previous variable-reward-condition trial (Figure 4Biii) (*p* = 1.05E-9 and 0.00144), the condition interference in fixed-reward condition was weak as compared to that in the variable-reward condition. Taken together, these results indicate that flexible behaviors are more likely affected by events in another condition than inflexible behaviors.

We further tested whether the differences in choice probabilities were observed by simply ignoring the trial condition. Supplementary Figure 1 shows the conditional choice probabilities in the variable-reward condition. We found that the experience of optimal choice rewarded in the fixed-reward condition affected the choices in the subsequent variable-reward condition significantly less than that in the variable-reward condition did. This weak effect of the fixed-reward condition indicates the existence of condition interference, while rats did not completely ignore the conditions.

#### Model-based analysis

**Figure 5:**
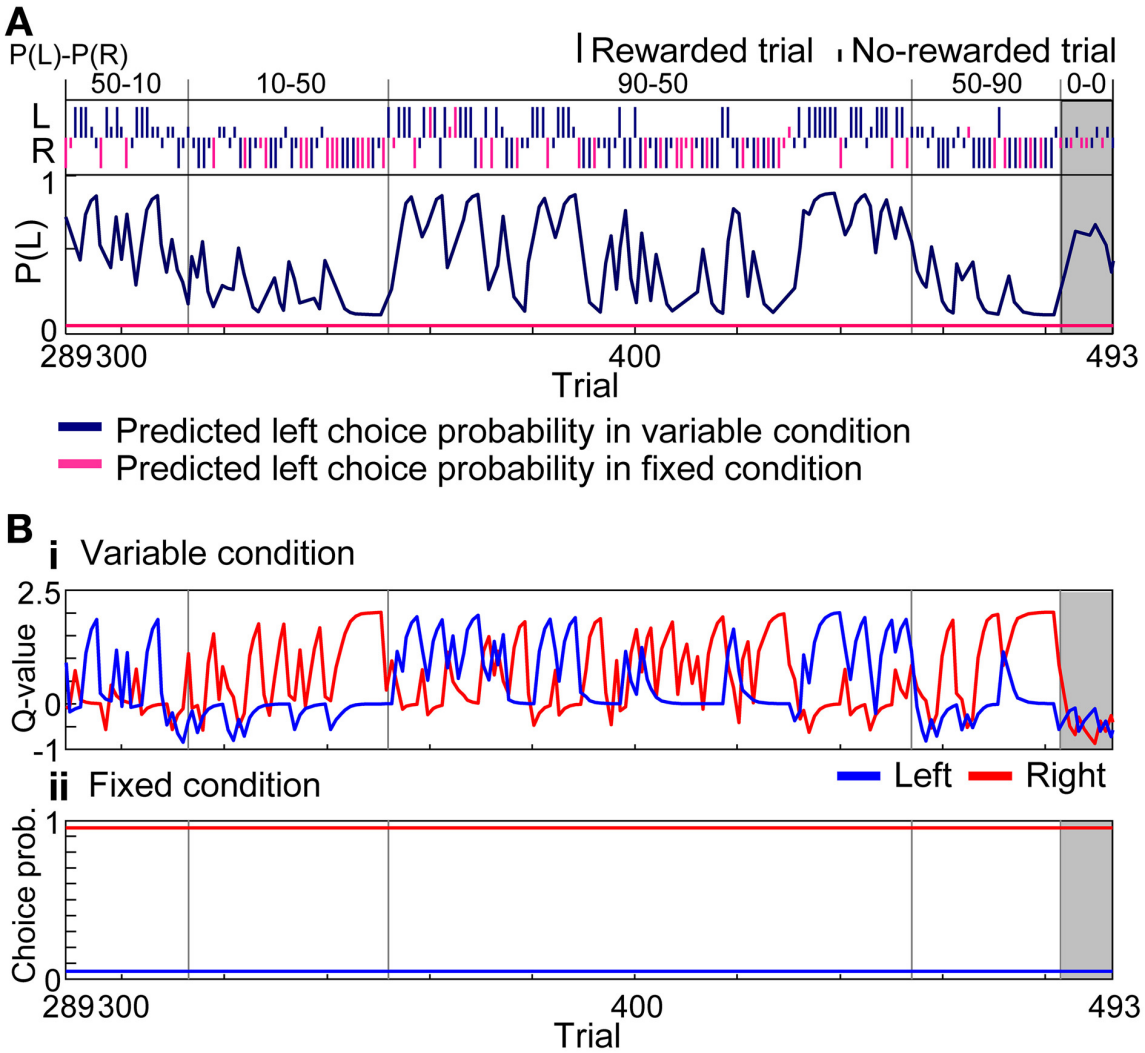
**Learning interference model. (A)** Prediction of choice probability. The learning interference model predicted choice behaviors of rat in Figure 3 between the 289 and 493 trials. Trials consisted of all four reward-probability settings in the variable-reward condition and the extinction phase. FQ-learning and fixed-choice models were employed as learning rules of variable- and fixed-reward conditions, respectively. Free parameters of the learning interference model were set to achieve maximum likelihood in this session. Dark blue and pink lines show predicted left choice probabilities of the model in variable- and fixed-reward conditions, respectively. Other lines and symbols are as in Figure 3. The learning interference model accurately predicted the choice behaviors of rat. **(B)** Model variables. **(i)** Action values of the variable-reward condition were predicted by FQ-learning. **(ii)** Choice probabilities in the fixed-reward condition were predicted by the fixed-choice model.

To quantify the interference in variable- and fixed-reward conditions, we analyzed choice behaviors with reinforcement learning models. Figure 5A modeled the choices of rat in Figure 3 with the learning interference model in which FQ-learning (i.e., a modified Q-learning) and fixed-choice model, assuming constant action values, were used to update values of the variable- and fixed-reward conditions, respectively (see Materials and Methods). In variable-reward-condition trials, the FQ-learning captured the quick change of choices, while, in fixed-reward-condition trials, the fixed-choice model captured the continuous selection of the optimal choice. Action values of the variable-reward condition were updated in both variable-reward-condition and fixed-reward-condition trials, and a quick change of values predicted rapid choice changes in the variable-reward condition (Figure 5B). In contrast, the fixed choice probability for the fixed-reward condition predicted inflexible behaviors.

**Figure 6:**
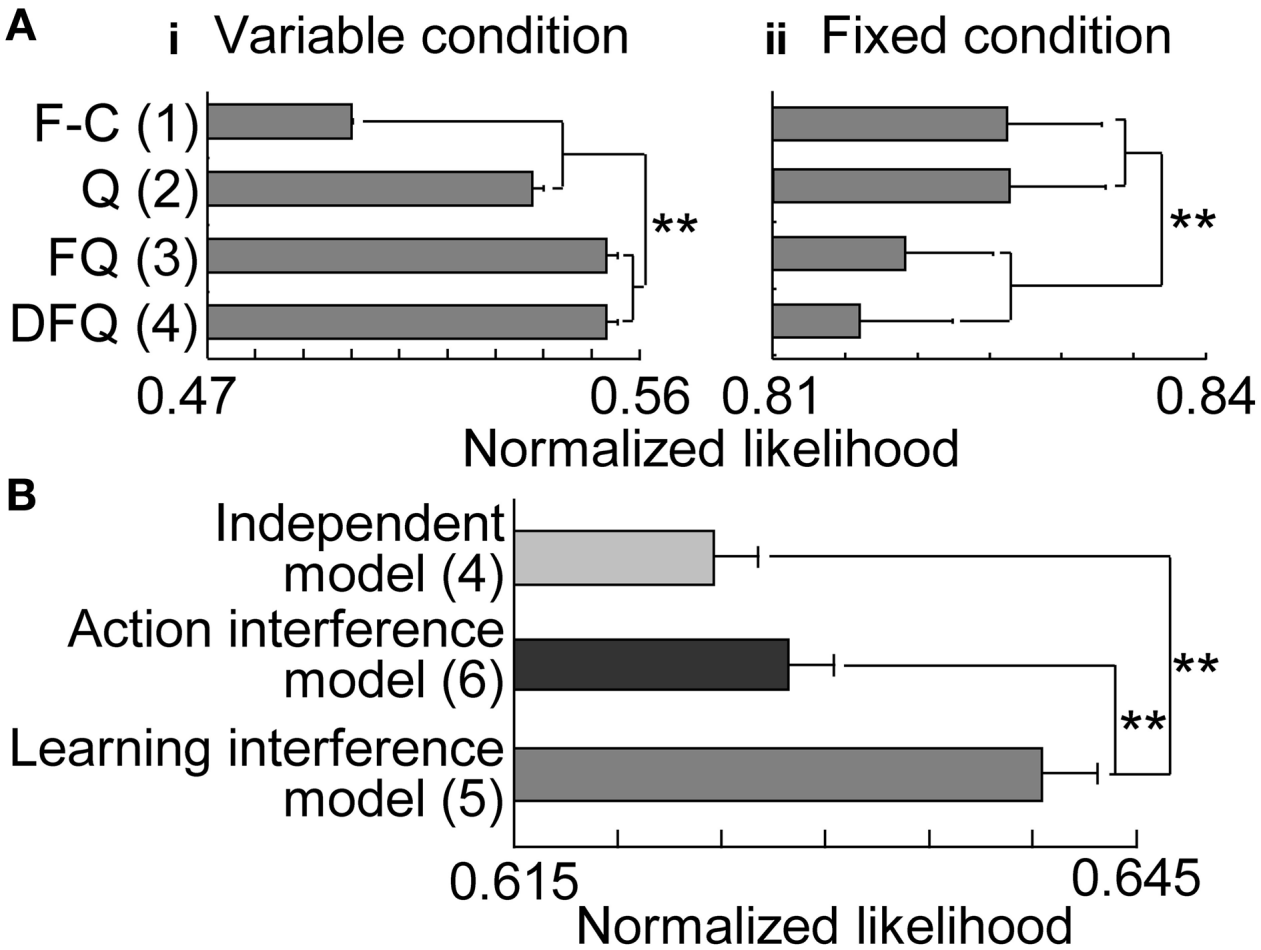
**Model fitting. (A)** Trials with variable- **(i)** and fixed-reward conditions **(ii)**. Results of 2-fold cross validation were compared among the four models: F-C, fixed-choice model; Q: standard Q-learning; FQ: forgetting Q-learning; DFQ: differential forgetting Q-learning. Means and standard errors are shown. The number of free parameters in each model is shown in parentheses: ^**^p < 0.01 in a two-sided paired *t*-test. **(B)** All trials. FQ-learning and fixed-choice models were employed as learning rules of variable- and fixed-reward conditions, respectively.

First, we separately fit reinforcement learning models to the choices in variable- and fixed-reward conditions and analyzed normalized likelihoods in 2-fold cross validation (Figure 6A). In the variable-reward condition, FQ-learning and DFQ-learning better fit the behaviors than the Q-learning and fixed-choice models (two-sided paired *t*-test, *p* = 3.95E-26 − 1.41E-44), while the results were completely opposite in the fixed-reward condition (*p* = 6.63E-6 − 4.33E-8), indicating that the choice strategy depended on the condition. Based on the results, we employed the FQ-learning and fixed-choice models for the learning rules of variable- and fixed-reward conditions, respectively. We then compared normalized likelihoods among the independent model, the action interference model, and the learning interference model (Figure 6B), to test whether condition interference happened in the action selection or value updating phase. The learning interference model better fit the choice behaviors of rats than did other models (*p* = 4.91E-22 and 1.34E-28), and this significant trend was the same when DFQ-learning and Q-learning were employed as the learning rules of variable- and fixed-reward conditions, respectively (*p* = 2.84E-8 − 1.94E-28). In the learning interference model, FQ-learning for the variable-reward condition updated the values with the events in both the variable-reward-condition and fixed-reward-condition trials, while the fixed-choice model for fixed-reward condition had a fixed-choice probability in all trials (see Materials and Methods). Thus, these results indicate that (i) condition interference occurred in the value updating phase, (ii) the choices in variable-reward condition were affected adversely by events in the fixed-reward condition, and (iii) choices in the fixed-reward condition were not affected by events in either the variable- or fixed-reward conditions.

In the learning interference model, the degree of condition interference was captured by the free parameters, i.e., learning rates. We set the free parameters to achieve the maximum likelihood in each session; in variable-reward condition, the learning rate for updating values with events in the variable-reward condition (α_1, *V*, *V*_) was 0.734 ± 0.0192, while the learning rate with events in the fixed-reward condition (α_1, *F*, *V*_) was 0.432 ± 0.0206. This indicates that interference from the fixed-reward condition was weaker than learning from the variable-reward condition (Wilcoxon signed-rank test, *p* = 8.07E-20).

### Neural analysis

**Figure 7:**
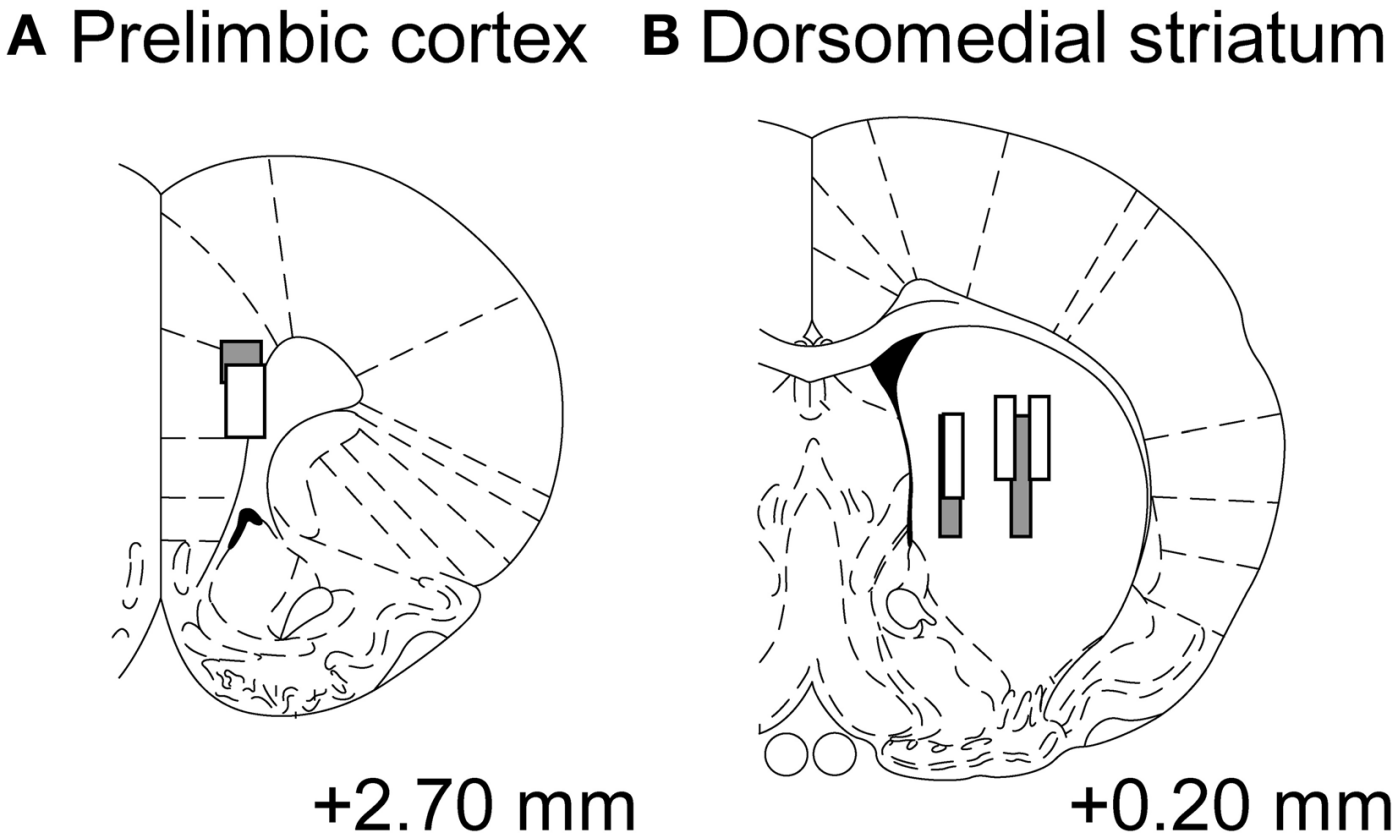
**Tracks of electrode bundles**. Each diagram shows a coronal section referenced to the bregma (Paxinos and Watson, 1997). Data recoding from the sites **(A,B)** were treated as neuronal activities from the prelimbic cortex and dorsomedial striatum, respectively. Gray-level of boxes distinguishes electrode tracks from each rat.

We recorded neural activities from 2 rats during 19 sessions in total, and recorded from 26 neurons (rat 3: 24, rat 5: 2) in the prelimbic cortex and 26 neurons (rat 3: 6, rat 5: 20) in the dorsomedial striatum. Some neurons were recorded from a slightly central part of the dorsal striatum, but we analyzed them as the part of dorsomedial striatum (Figure 7) (Stalnaker et al., 2010; Wang et al., 2013).

#### Action, reward, and condition coding

**Figure 8:**
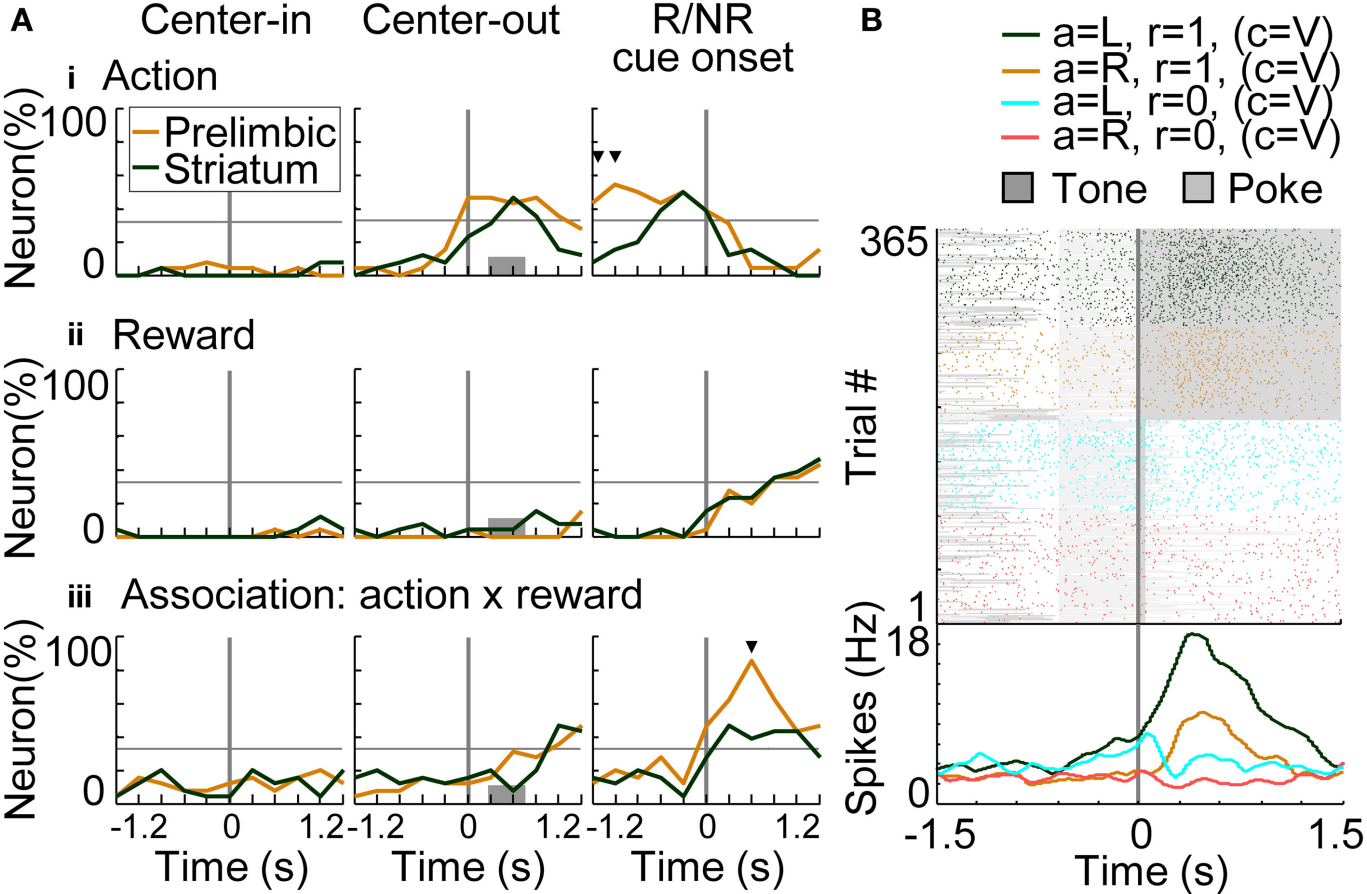
**Coding of response-outcome (R-O) association. (A)** The proportion of neurons coding actions (responses: R) **(i)**, rewards (outcomes: O) **(ii)** and R-O associations **(iii)**. In regression analysis with variable-reward-condition trials, the proportion of neurons with significant regression coefficients is shown: *p* < 0.01 in a two-sided Student's *t*-test. Orange and green lines indicate neurons in the prelimbic cortex and dorsomedial striatum, respectively. When the proportion of neurons exceeded the threshold (32.3%), we defined that the prelimbic cortex or the striatum significantly represented the variable (*z*-test, *p* < 0.05). In each column, proportions of neurons are aligned with a different timing: left, initiation of center-hole poking; middle, end of center-hole poking; right, onset of reward or no-reward cue (R/NR cue). Gray bars in the middle column show the timing of right- or left-hole poking in 99.3% of all trials. Small black triangles indicate that the proportions of neurons in the prelimbic cortex and dorsomedial striatum had significant differences: *p* < 0.01 in a χ^2^-test. **(B)** Representative prelimbic cortical neuron encoding the R-O association. Neural activities at the onset of reward or no-reward cues in the variable-reward condition are shown, as in the right column of **(A)**. Green and orange colors show left and right choices in rewarded trials, respectively. Light blue and red colors show non-rewarded trials. Raster plot: colors of spikes differ with actions and outcomes of trials. Tone presentations and poking periods are shown with gray boxes. The lower part shows the average spike density function smoothed with a Gaussian kernel with a standard deviation of 50 ms.

To investigate temporal dynamics of neural coding in the prelimbic cortex and dorsomedial striatum, regression analyses were conducted with a time window of 600 ms advanced with a time step of 300 ms. Figure 8A shows results of regression analysis in the variable-reward condition for investigating the coding of actions (i.e., responses: R), rewards (i.e., outcomes: O), and R-O associations (Equation 7). When the number of neurons encoding each variable exceeded the threshold of 32.3% (9 out of 26 neurons), we determined that the prelimbic cortex or dorsomedial striatum significantly encoded the variable (*z*-test, *p* < 0.05). In action coding, both prelimbic and striatal neurons participated significantly (46.2%, *z*-test, *p* = 0.00924) (Figure 8Ai at the middle column). Prelimbic neurons encoded actions during choice timing, while striatal neurons encoded them only after the choice, suggesting that action execution was represented in the prelimbic cortex. Prelimbic and striatal neurons equally and significantly represented rewards after the reward or no-reward cue. At this cue timing, more prelimbic than striatal neurons encoded R-O associations (χ^2^-test, *p* = 6.25E-4) (Figure 8Aiii). A representative prelimbic neuron increased activities only after the reward tone at left choice (Figure 8B).

**Figure 9:**
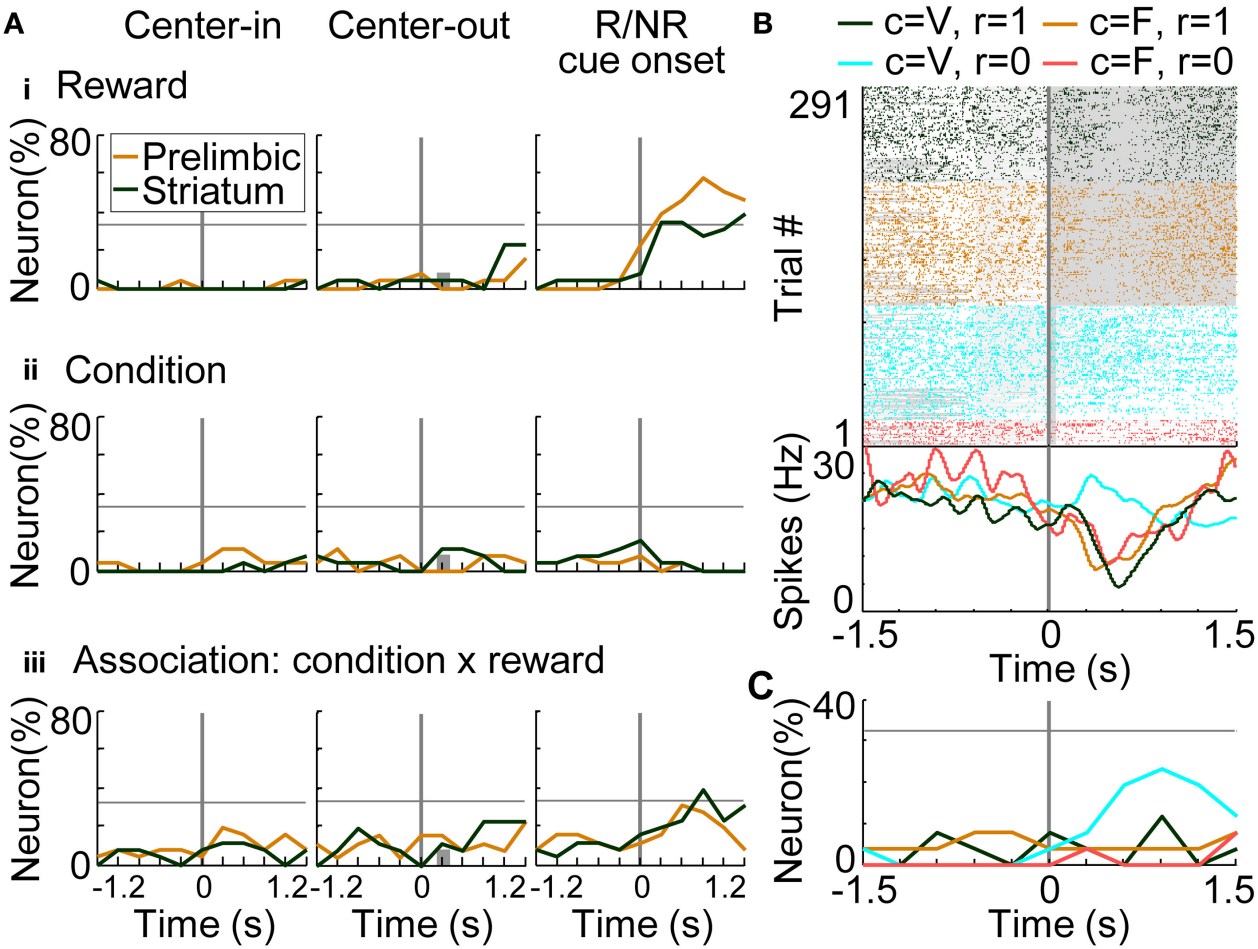
**Coding of stimulus-outcome (S-O) association. (A)** Proportions of neurons coding rewards (outcomes: O) **(i)**, conditions (stimuli: S) **(ii)** and S-O associations **(iii)**. Regression analysis was performed on data from trials in which rats made a choice to the optimal side of fixed-reward condition. The proportion of neurons that had significant regression coefficients is shown: *p* < 0.01 in a two-sided Student's *t*-test. Lines and symbols as in Figure 8A. **(B)** Representative dorsomedial striatal neuron encoding the S-O association. Neural activities at the onset of reward or no-reward cues are shown, as in the right column of **(A)**. Green and orange colors show activities in rewarded trials in the variable- and fixed-reward conditions, respectively. Light blue and red colors show non-rewarded trials. Lines and symbols as in Figure 8B. **(C)** Detailed neural coding of S-O associations in the dorsomedial striatum. The proportion of neurons encoding one of the four associations is shown before and after the onset of reward or no-reward cues, as in **(B)**. Colors correspond to **(B)**. Many striatal neurons encoded the no-reward cue in the variable-reward condition, indicating that they did not differentiate reward and no-reward cues in the fixed-reward condition.

For investigating the coding of conditions (i.e., stimuli: S) and associations between conditions-rewards [i.e., stimuli-outcomes (S-O)], we conducted regression analysis on the trials in which rats selected the optimal side of the fixed-reward condition (Equation 8). There was no significant difference in the number of prelimbic cortical and dorsomedial striatal neurons that functioned as reward-coding neurons (Figure 9Ai), consistent with results in the variable-reward condition (Figure 8Aii). In the prelimbic cortex and the striatum, the number of condition-coding neurons did not reach the significant level in our sample (32.3% for *n* = 26) (Figure 9Aii). The number of neurons encoding S-O associations was significant only in the striatum (38.5%; *z*-test, *p* = 0.0248) (Figure 9Aiii). A representative striatal neuron increased activity only at the no-reward cue in variable-reward condition (Figure 9B). Overall dorsomedial striatal neurons mainly represented the no-reward cue in variable-reward condition, suggesting that they differentiated and ignored the outcomes in variable- and fixed-reward conditions, respectively (Figure 9C).

#### Value coding

**Figure 10:**
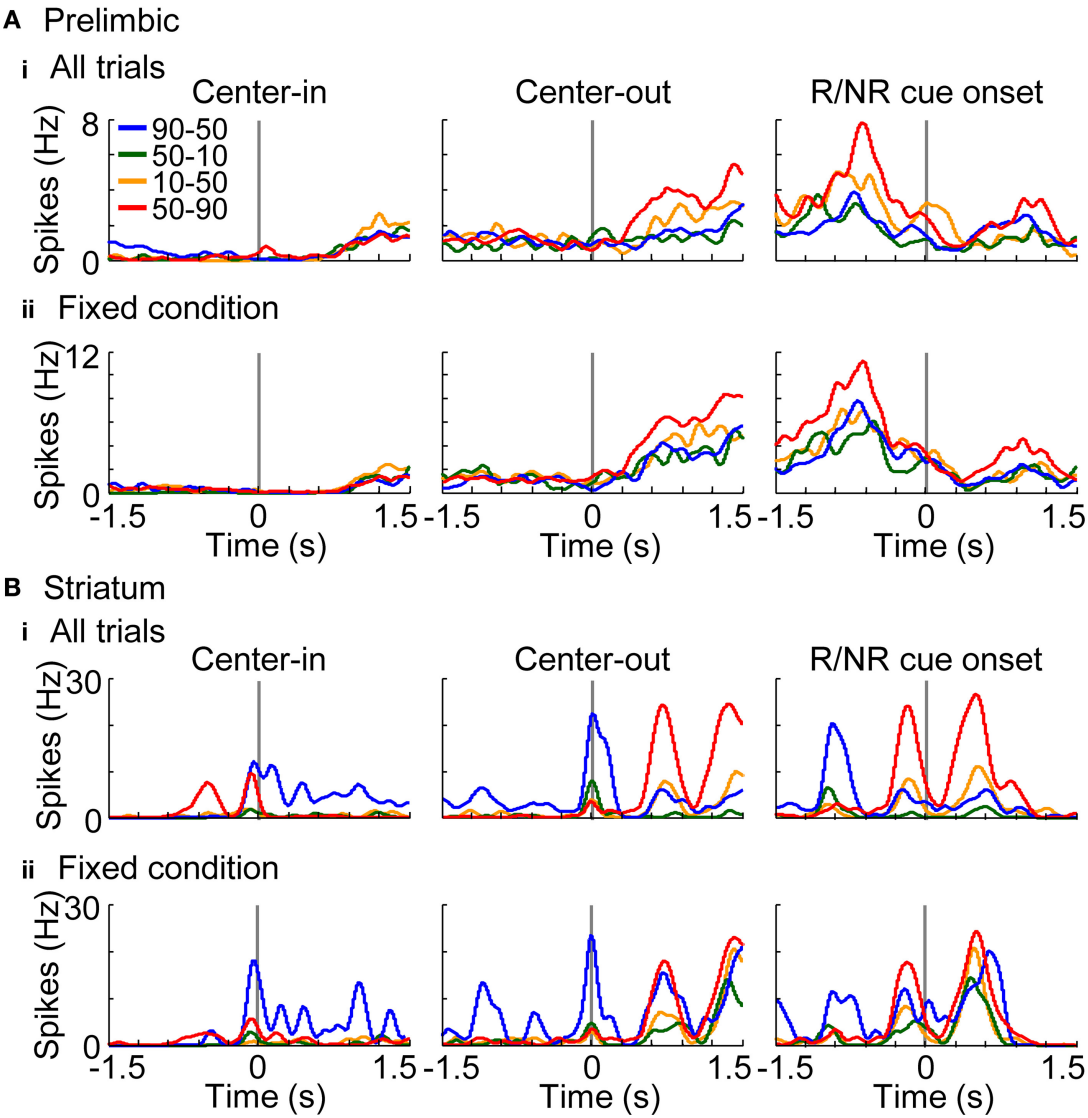
**Representative value-coding neuron**. Representative neurons coding values of the variable-reward condition are shown from the prelimbic cortex **(A)** and dorsomedial striatum **(B)**. Average spike density functions during both the variable-reward-condition and fixed-reward-condition trials **(i)** and during the fixed-reward-condition trials **(ii)** are shown, smoothed with a Gaussian kernel with a standard deviation of 50 ms. Each colored line shows the activity during a reward-probability block in the variable-reward condition; reward probabilities for left-right choices are shown in the inset. Activities are aligned with different timings as in Figure 8A. Prelimbic cortical neuron represented state values after the center-hole poking **(A)**, while dorsomedial striatal neuron represented state and action values during and after the center-hole poking, respectively **(B)**. Both neurons represented values of the variable-reward condition even during fixed-reward-condition trials **(ii)**.

In addition to elucidating neural coding of basic task features (i.e., actions, rewards, and conditions), investigations of the coding of decision variables (values) are important for understanding learning algorithms of rats. Values were derived from the learning interference model which achieved the highest normalized likelihood among the models (Figure 6B). Free parameters of the model were set to achieve the maximum likelihood in each session. Figures 10A,B show a representative value-coding neuron in the prelimbic cortex and dorsomedial striatum, respectively. Prelimbic neuron encoded state values of the variable-reward condition after the center-hole poking; value coding was observed even during the fixed-reward-condition trials (Figure 10Aii). Values of the variable-reward condition were updated both with events in variable- and fixed-reward conditions with a forgetting effect, such that the state value was high when the reward probability of variable- and fixed-reward conditions were identical (Figure 10A). Striatal neuron in Figure 10B also represented state values and action values of the variable-reward condition in fixed-reward-condition trials during and after the center-hole poking, respectively. These results show that neurons in the prelimbic cortex and dorsomedial striatum represent and store values of the variable-reward condition.

**Figure 11:**
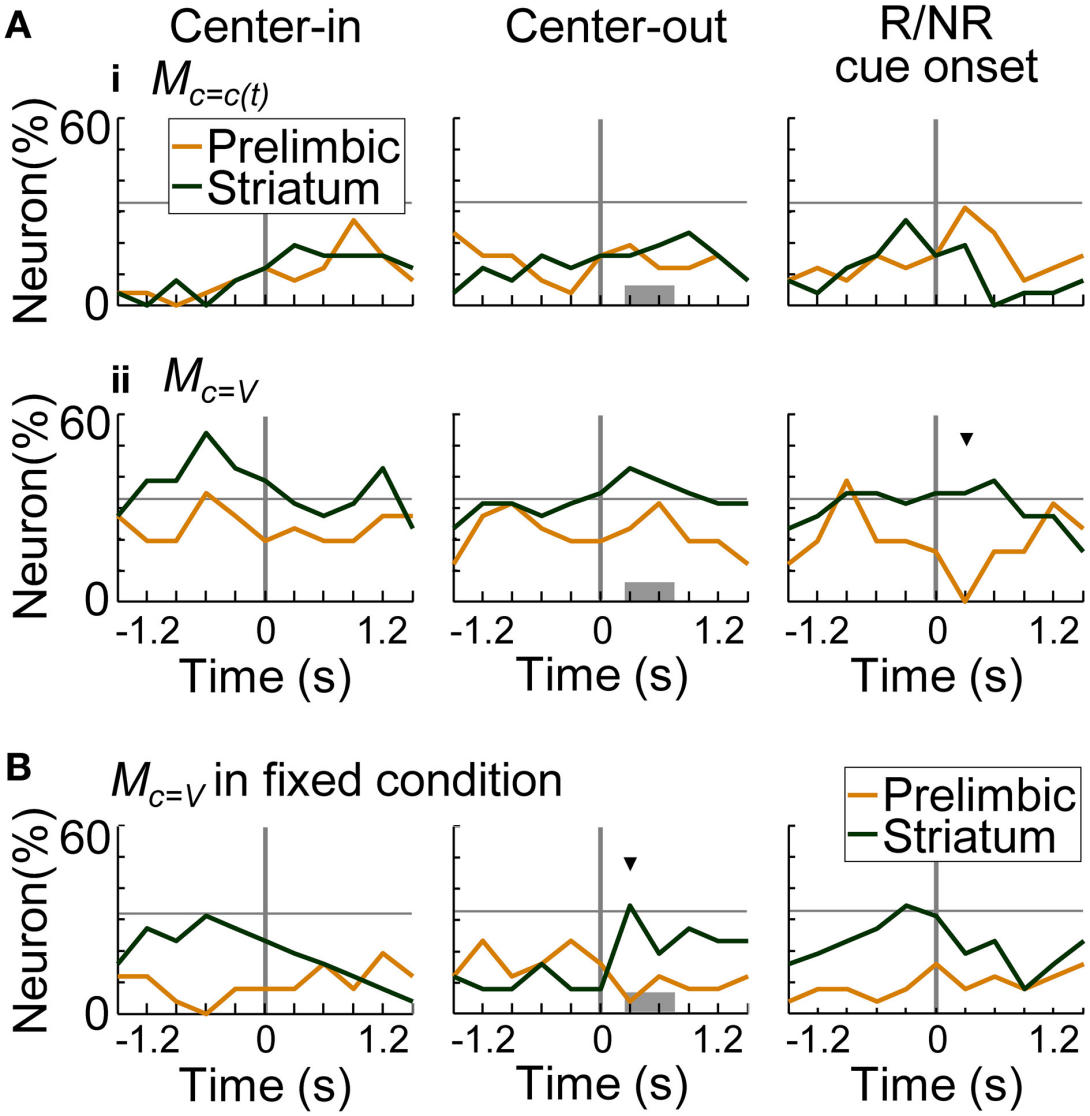
**Coding of values in variable-reward condition. (A)** The proportion of neurons coding the values of the presented-trial condition **(i)** and of the variable-reward condition **(ii)**. In regression analysis with all trials, the proportion of neurons which had significant regression coefficients was shown: *p* < 0.01 in a two-sided Student's *t*-test. Values consisted of five variables (i.e., action values for left and right choices, state value, chosen value, and policy) in the learning interference model. Lines and symbols as in Figure 8A. Dorsomedial striatal neurons mainly encoded values of the variable-reward condition. **(B)** The proportion of neurons encoding values of the variable-reward condition in fixed-reward-condition trials. Neurons representing values of the variable-reward condition were investigated with regression analysis using fixed-reward-condition trials. Lines and symbols as in **(A)**.

Figure 11A summarizes the proportion of neurons encoding values of the presented-trial condition (i) and of the variable-reward condition (ii). Striatal neurons significantly encoded primarily values of the variable-reward condition (*z*-test, *p* < 0.05). Especially after a reward or no-reward cue, a larger proportion of neurons in the striatum encoded the values than in the prelimbic cortex (χ^2^-test, *p* = 9.70E-4) (Figure 11Aii at the right column). Moreover, even during fixed-reward-condition trials, striatal neurons encoded values of the variable-reward condition after the center-hole poking (34.6%; *z*-test, *p* = 0.0388) (Figure 11B at the middle column). These results suggest that dorsomedial striatal neurons track values for flexible behaviors.

## Discussion

In this study, we used rats to conduct a free choice task with a random trial sequence of variable- and fixed-reward conditions, and recorded neuronal activity in the prelimbic cortex and dorsomedial striatum. In variable- and fixed-reward conditions, rats displayed flexible and inflexible choice behaviors, respectively, which were tested with an extinction phase (Figure 4A). We then observed some interference between the behaviors. When holes with a higher reward probability in variable-reward-condition and fixed-reward-condition trials were different, rats took more trials to find the better hole in the variable-reward condition, compared to when the holes were identical. In addition, choices in the variable-reward condition were affected by previous fixed-reward-condition trials, while choices in the fixed-reward condition were relatively stable (Figure 4B). Thus, flexible behaviors are more likely to be affected by events in another condition than are inflexible behaviors. Our reinforcement learning models suggest that condition interference happens in the value-updating phase (Figure 6B). Based on the following observations, condition interference is likely distinct from ignoring the trial condition. First, rats successfully selected the more-rewarding holes in the variable-reward condition in all reward-probability settings, while they could keep selecting the optimal choice for the fixed-reward condition (Figure 3). Second, action-outcome experiences in variable- and fixed-reward conditions had different effects on subsequent choices in the variable-reward condition (Supplementary Figure 1). Third, reinforcement learning models showed that, in the variable-reward condition, learning from the fixed-reward condition was weaker than that from the same condition.

Some prelimbic cortical and dorsomedial striatal neurons associated actions with rewards irrespective of trial conditions (Figures 8A, 9A). Prelimbic and striatal neurons were likely to track values of the variable-reward condition, but not values of the on-going fixed-reward condition (Figures 10, 11A). We then verified that some striatal neurons tracked values of the variable-reward condition even during fixed-reward-condition trials (Figure 11B), such that values were updated irrespective of trial conditions. This was possibly utilized in the learning-interference reinforcement-learning model and caused an interfering value-updating in variable-reward condition.

### Interference reinforcement learning models

Variable- and fixed-reward conditions are considered independent states in reinforcement learning theory (Sutton and Barto, 1998; Dayan and Niv, 2008), so the optimal action in each condition was independently determined. Conventional reinforcement learning algorithms usually aim to find an optimal action in each condition and do not consider conditional relationships. However, humans and animals have dependencies among conditions. For example, monkeys and rats decide actions based on reward experiences in other conditions (Balleine and Dickinson, 1998; Gallagher et al., 1999; Balleine, 2005; West et al., 2011; Jones et al., 2012). With such knowledge transfers, a condition interference is also reported; humans cannot perform two tasks at once without a delay in reaction time (Monsell, 2003). Our results also clearly showed interference in the variable-reward condition (Figure 4B).

Condition interference was considered in our proposed reinforcement learning models. The learning interference model assumed that interference occurred in the value updating phase, and that learning efficacy should be different between learning from its own condition and from other conditions (Suzuki et al., 2012). In contrast, the action interference model assumed that there was interference in the action selection phase instead. In the action selection phase, the model utilized the action values of other conditions as accelerators or inhibitors of action.

To purely test condition interference, the learning rule in each condition should be properly selected. In addition to the three varieties of reinforcement learning models in Ito and Doya (2009), we employed a fixed-choice model. The model had a constant choice probability, assuming the zero learning rate or the completion of learning.

### Learning algorithms of flexible and inflexible behaviors

Consistent with previous findings with rats (Ito and Doya, 2009; Funamizu et al., 2012), FQ- or DFQ-learning fit the flexible choice behaviors of rats in the variable-reward condition (Figure 6A). On the other hand, in the fixed-reward condition, standard Q-learning or the fixed-choice model fit the inflexible behaviors, suggesting that flexible and inflexible behaviors have different learning algorithms. No forgetting of action values or no learning in Q-learning or the fixed-choice model left the choice prediction constant, compared to FQ- or DFQ-learning, and such no learning was observed in the extinction phase in the fixed-reward condition (Figure 4A). One possible reason for the strategy difference is that rats reduced the costs and times of inflexible behaviors, since Q-learning and fixed-choice models had simpler value-updating rules than FQ- and DFQ-learning (Equation 3).

The condition interference in flexible and inflexible behaviors was quantitatively tested with the learning interference model (Figure 6B). In this model, the fixed-choice model in the fixed-reward condition provided the constant choice probability, and thus predicted no interference. On the other hand, FQ-learning in the variable-reward condition updated action values with events in both variable- and fixed-reward conditions, suggesting the existence of interference.

### Neural coding in the prelimbic cortex and dorsomedial striatum

The prelimbic cortex and the dorsomedial striatum represented the response-outcome (R-O) association (Figure 8Aiii). The R-O association is essential for a goal-directed system, which is known to be driven by the aforementioned brain regions (Corbit and Balleine, 2003; Yin et al., 2005b; Balleine et al., 2007). Flexible behaviors in the variable-reward condition also required R-O associations for action learning: flexible behaviors and the goal-directed system may be related. Action or reward coding was also observed both in the prelimbic cortex and the dorsomedial striatum (Figures 8Ai,ii), consistent with recent studies (Kim et al., 2009; Sul et al., 2010).

In addition, a stimulus-outcome (S-O) association was observed in the dorsomedial striatum (Figure 9Aiii). This association is important to evaluate and differentiate between values of each condition and is essential for reward-based adaptive behaviors under multiple conditions. Especially in this study, striatal neurons evaluated and ignored outcomes of variable- and fixed-reward conditions, respectively (Figures 9B,C), supporting the formation of flexible and inflexible behaviors.

Such associative representations were mainly found after choices and reward cues in our study; at this time, value updating is required for deciding future actions. Value updating requires calculating a temporal difference error, which is known to be represented in midbrain dopaminergic neurons (Schultz et al., 1997; Schultz, 1998). Dopaminergic neurons mainly project to the striatum (Schultz, 1998) which represented S-O and R-O associations in the value-updating phase of our study (Figures 8, 9). Thus, the dorsomedial striatum is likely to play a role in associating the variable-reward condition with rewards, and actions with rewards, via dopamine-induced potentiation (Reynolds et al., 2001; Kim et al., 2009). In contrast, reward and no-reward events in the fixed-reward condition were ignored in some striatal neurons (Figures 9B,C), suggesting no value updating. The prelimbic cortex encoded R-O associations in the value-updating phase (Figure 8Aiii). Dopaminergic neurons also project to the frontal cortex (Schultz, 1998), implying that the prelimbic cortex contributes to memorization of rewarded actions (Euston et al., 2012).

Neural potentiation in R-O associations (Figure 8Aiii) or rewards (Figure 9Ai), irrespective of the trial condition, sometimes might facilitate suboptimal action, especially when more rewarding choices of variable- and fixed-reward conditions are different. This effect was actually seen in the choice behaviors of rats: the number of trials required to select the better-rewarding option in the variable-reward condition was significantly larger when optimal choices of both conditions were different than when the choices were identical. Thus, neural coding in the prelimbic cortex and dorsomedial striatum predicted condition interference.

### Coding of values in variable-reward condition

The prelimbic cortex and dorsomedial striatum mainly encoded values of the variable-reward condition, and not of the condition in the on-going trial (Figures 10, 11). In our task, rats needed to keep tracking values of the variable-reward condition to make the optimal choice, even in fixed-reward-condition trials, and the value tracking might be observed as the activities. The prefrontal cortex has the ability to track a value of an unchosen option (Boorman et al., 2009). The dorsomedial striatum is also known to be involved in long-term retention (El Massioui et al., 2007), supporting a hypothesis that the prelimbic cortex and dorsomedial striatum can represent the values of un-experiencing actions or conditions. Such value representations possibly generated the interfering behaviors of rats (Figure 6B). Values in the variable-reward condition were encoded before rats knew the trial condition, i.e., before center-hole poking (Figure 11, left column), suggesting that rats mainly prepared for the variable-reward condition, which was assigned in 70% of all trials.

## Conclusion

Our behavioral analyses with reinforcement learning models indicate that rats had an interfering value-updating in the variable-reward condition. Our electrophysiological study suggests that this interfering value-updating is mediated by the prelimbic cortex and dorsomedial striatum. First, although some dorsomedial striatal neurons represented condition-reward associations, the prelimbic cortex and striatum associated actions with rewards irrespective of trial conditions. Second, striatal neurons kept tracking values of the variable-reward condition even during the fixed-reward condition, such that values were possibly interferingly updated even in the fixed-reward condition.

### Conflict of interest statement

The authors declare that the research was conducted in the absence of any commercial or financial relationships that could be construed as a potential conflict of interest.

## Abbreviations

AP: anterior-posterior
dB SPL: sound pressure level in decibels
DFQ-learning: differential forgetting Q-learning
FQ-learning: forgetting Q-learning
ML: medio-lateral
O: outcome
R: response
S: stimulus.
