# Supplementary material for: Condition interference in rats performing a choice task with switched variable- and fixed-reward conditions
Source: Front Neurosci. 2015 Feb 13;9:27. doi: 10.3389/fnins.2015.00027 (PMC4327310; doi:10.3389/fnins.2015.00027)
Supplement: Supplementary file 1 [file DataSheet1.DOCX]

***Supplementary Material***

**Condition interference in rats performing a choice task with switched variable- and fixed-reward conditions**

**Akihiro Funamizu^1,2^*, Makoto Ito^1^, Kenji Doya^1^, Ryohei Kanzaki^2,3^, Hirokazu Takahashi^2,3^**

^1^ Neural Computation Unit, Okinawa Institute of Science and Technology Graduate University, 1919-1, Tancha, Onna-son, Kunigami, Okinawa 904-0495, Japan

^2^ Graduate School of Information Science and Technology, The University of Tokyo, Hongo 7-3-1, Bunkyo-ku, Tokyo 113-8656, Japan

^3^ Research Center for Advanced Science and Technology, The University of Tokyo, Komaba 4-6-1, Meguro-ku, Tokyo 153-8904, Japan

*** Correspondence:** Akihiro Funamizu, Ph.D., Neural Computation Unit, Okinawa Institute of Science and Technology Graduate University, 1919-1, Tancha, Onna-son, Kunigami, Okinawa 904-0495, Japan.

funamizu@oist.jp

1. **Supplementary Figure**


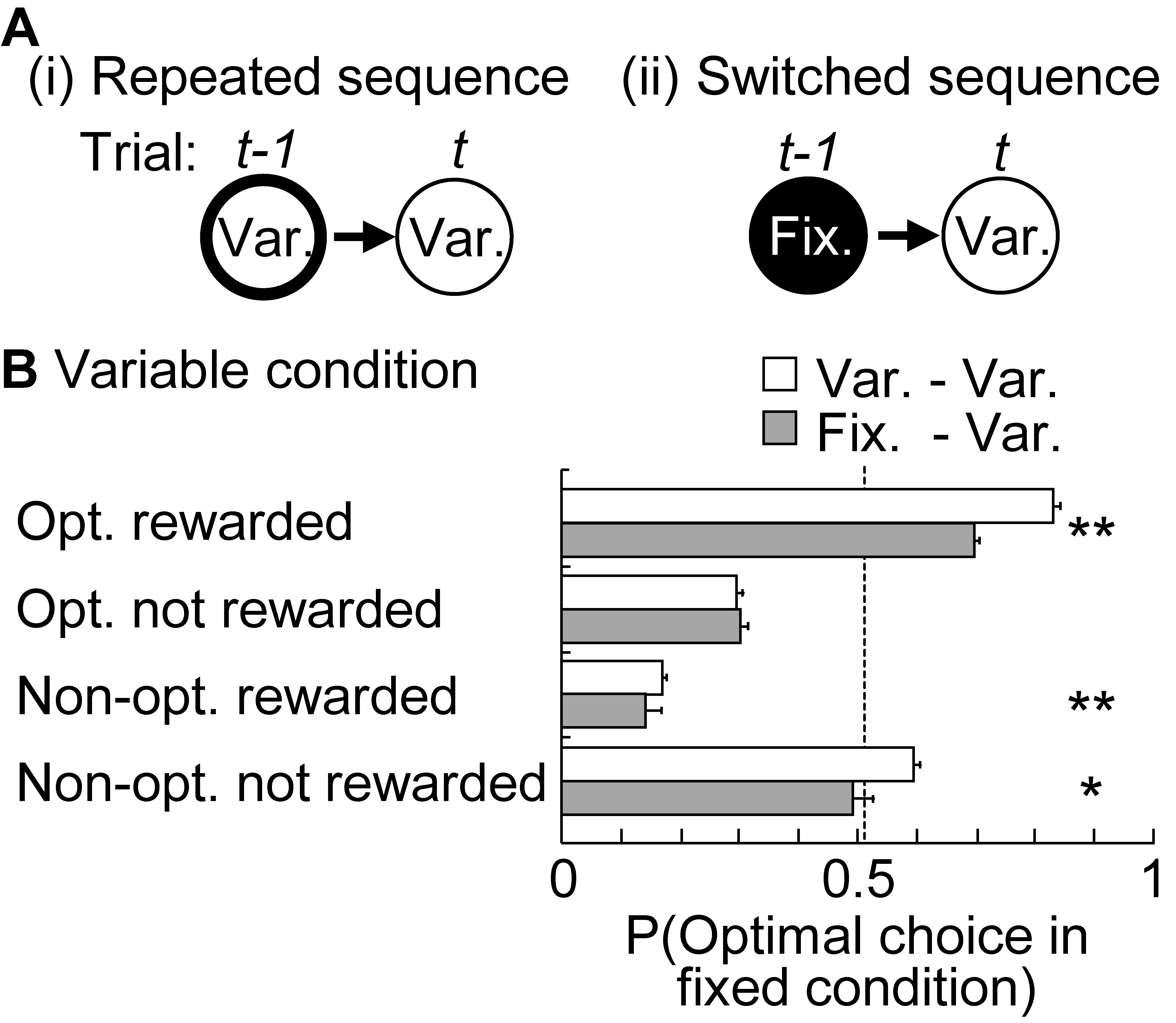


Supplementary Figure 1. Detailed analysis of condition interference in variable-reward condition

(A) Calculation of conditional choice probability. The conditional probability at trial *t* with the variable-reward condition (Var.) was analyzed based on the action-outcome experience at trial *t*-1 with the variable- (Var.) and fixed-reward conditions (Fix.) in (i) and (ii), respectively. Action-outcome experiences were of 4 types: optimal choice rewarded (Opt. rewarded); optimal choice not rewarded (Opt. not rewarded); non-optimal choice rewarded (Non-Opt. rewarded); non-optimal choice not rewarded (Non-opt. not rewarded). If the condition of trial *t*-1 was ignored, conditional choice probabilities in Var. - Var. and Fix. - Var. sequences should be the same.

(B) Comparison of conditional choice probabilities between Var. - Var. and Fix. - Var. sequences. Means and standard errors of probabilities are shown. Dotted line shows the average choice probability. If rats perfectly distinguished the variable- and fixed-reward conditions, conditional probabilities of Fix. - Var. sequences (gray bars) should become the same in all action-outcome experiences. On the other hand, if rats ignored the trial conditions, conditional probabilities in Var. - Var. and Fix. - Var. sequences should be the same. Our results show that neither case was likely: gray bars varied depending on the action-outcome experiences, and the white and gray bars had significant differences under some experiences. These results indicate that rats distinguished the trial condition with some interference, but they could utilize experiences of variable- and fixed-reward conditions differently: *, p < 0.05; **, p < 0.01 in a Mann-Whitney U-test.
